# Associations between uncertainty in illness and cardiac rehabilitation adherence in patients with CHD: a cross-sectional chain mediation and latent profile analysis of cardiac self-efficacy and fear of activity

**DOI:** 10.3389/fcvm.2026.1846950

**Published:** 2026-08-19

**Authors:** Lei Wu, Hongmei Xue, Chunxiu Tian, Yan Lu

**Affiliations:** 1 Department of Cardiology, First Hospital of Shanxi Medical University, Taiyuan City, Shanxi Province, China; 2 Department of Cardiology, Fenyang Hospital of Shanxi Province, Fenyang City, Shanxi Province, China; 3 Department of Cardiology, Changzhi Medical College Affiliated Hospital, Changzhi People’s Hospital, Changzhi, Shanxi Province, China

**Keywords:** cardiac rehabilitation adherence, cardiac self-efficacy, coronary heart disease, fear of activity, uncertainty in illness

## Abstract

**Objective:**

This study aimed to integrate variable-centered and person-centered approaches to examine the relationship between uncertainty in illness and cardiac rehabilitation adherence in patients with coronary heart disease (CHD), as well as the underlying psychological mechanisms involved. In addition, the study sought to identify latent subgroups of uncertainty in illness and to compare differences across these subgroups.

**Methods:**

A cross-sectional study was conducted from August 2024 to April 2025. Patients with CHD were recruited from the departments of cardiology and cardiac rehabilitation centers of three hospitals in Shanxi Province, China.

**Results:**

In the variable-centered analysis, uncertainty in illness was significantly and negatively associated with cardiac rehabilitation adherence, with a direct association coefficient of −0.119. Cardiac self-efficacy and fear of activity were involved in a statistically significant sequential indirect association. In the person-centered analysis, latent profile analysis identified three subgroups of uncertainty in illness: a cognitively clear–low uncertainty group (26.27%), a partially informed–moderate uncertainty group (39.41%), and a cognitively ambiguous–high uncertainty group (34.32%). Significant differences were observed among the three subgroups in cardiac self-efficacy, fear of activity, and cardiac rehabilitation adherence.

**Conclusion:**

Clinicians should systematically assess the level of uncertainty in illness among patients with CHD at the initial stage of cardiac rehabilitation and develop tailored, stratified intervention strategies for different subgroups. In particular, patients classified in the cognitively ambiguous–high uncertainty group should be provided with comprehensive informational support, self-efficacy enhancement training, and cognitive-behavioral interventions targeting fear of activity, so as to effectively improve cardiac rehabilitation adherence in this population.

## 1 Introduction

Coronary heart disease (CHD) is caused by the accumulation of atherosclerotic plaques in the coronary arteries, resulting in vascular lumen narrowing or occlusion and thereby disrupting the balance between myocardial oxygen supply and demand, ultimately leading to myocardial ischemia, injury, or necrosis (1, 2). The number of patients with CHD in China has exceeded 11.39 million (3), and the prevalence continues to rise. Previous studies have shown that revascularization procedures, such as percutaneous coronary intervention and coronary artery bypass grafting, have substantially reduced acute-phase mortality in patients with CHD (4, 5). However, the long-term rehabilitation needs of these patients after treatment have not received sufficient attention. More importantly, cardiac rehabilitation encompasses multiple intervention components, including exercise training, health education, and psychological support, and has been shown to effectively reduce the recurrence of cardiovascular events, improve cardiopulmonary function, and enhance quality of life in patients with CHD (6, 7). Nevertheless, the clinical benefits of cardiac rehabilitation depend heavily on sustained and long-term patient participation (8). Cardiac rehabilitation adherence is commonly understood as the extent to which patients follow professional recommendations related to cardiac rehabilitation (9). However, its operational meaning may vary across studies, ranging from objective indicators such as attendance at supervised rehabilitation sessions to self-reported engagement in recommended rehabilitation behaviors. In the present study, cardiac rehabilitation adherence was specifically operationalized as patients' self-reported multidimensional adherence readiness and perceived implementation of prescribed or recommended cardiac rehabilitation behaviors after referral to, or during participation in, a cardiac rehabilitation program. This construct included five domains: exercise adherence, medication adherence, risk factor management adherence, nutritional management adherence, and psychological management adherence. Regrettably, previous studies have shown that cardiac rehabilitation adherence remains generally poor in clinical practice (1). This not only directly compromises the expected rehabilitation benefits, but is also closely associated with a higher incidence of adverse cardiovascular events and an increased risk of rehospitalization (10). Therefore, it is of considerable importance to explore the factors influencing cardiac rehabilitation adherence in patients with CHD and the mechanisms through which these factors operate.

From a clinical perspective, the persistence of poor adherence suggests that simply providing rehabilitation prescriptions, health education, or follow-up reminders may be insufficient to ensure sustained patient engagement. In routine cardiovascular rehabilitation practice, intervention programs are often delivered in a relatively standardized manner, with less attention paid to differences in patients' psychological responses to illness and rehabilitation. However, patients who receive similar rehabilitation recommendations may show markedly different behavioral responses: some actively participate and persist, whereas others hesitate, avoid exercise, or discontinue rehabilitation because of uncertainty, low confidence, or fear of symptom recurrence. This indicates that poor adherence may not only reflect insufficient knowledge or limited access to rehabilitation resources, but also the failure to identify and address distinct psychological phenotypes that shape patients' readiness and capacity to engage in rehabilitation. Therefore, clarifying the psychological mechanisms underlying cardiac rehabilitation adherence is clinically important for developing more precise and tiered intervention strategies.

However, cardiac rehabilitation adherence is unlikely to be shaped by a single risk factor or to manifest uniformly across all patients with CHD. As a complex health behavior, adherence is influenced by the joint effects of illness-related cognition, emotional responses, perceived behavioral capability, and contextual conditions. Consequently, the same level of exposure to cardiac rehabilitation information may lead to markedly different adherence patterns across individuals. This heterogeneity suggests that, beyond examining average associations among variables, it is also necessary to identify whether distinct psychological subgroups exist within the CHD population.

Uncertainty in illness is an important factor influencing health behaviors and rehabilitation outcomes in patients with chronic diseases (11, 12). Specifically, uncertainty in illness refers to a cognitive state that arises when individuals are unable to assign definite meaning to illness-related events (13). This state includes ambiguity regarding symptoms, unpredictability of disease progression, complexity of diagnostic and treatment-related information, and uncertainty about prognosis. During the course of treatment, patients with CHD often experience doubts about symptoms such as chest pain, concerns regarding the effectiveness of stent implantation or coronary artery bypass surgery, and uncertainty about rehabilitation regimens, all of which may further intensify their uncertainty in illness (14, 15). Previous studies have demonstrated that when patients appraise uncertainty in illness as a threat, it may trigger anxiety, avoidance oriented coping, and reduced engagement in health promoting behaviors (16, 17). Accordingly, high levels of uncertainty in illness may lead patients with CHD to question the safety and effectiveness of rehabilitation training, thereby diminishing their willingness to participate in and adhere to cardiac rehabilitation. In recent years, a growing body of evidence has confirmed a negative association between uncertainty in illness and adherence to health behaviors (18). For example, Papadakos, Hasan (19) found that patients with cancer who reported higher levels of uncertainty in illness tended to show poorer treatment adherence and lower engagement in self-management behaviors. Nevertheless, the clinical significance of this research gap extends beyond the limited number of prior studies. If the role of uncertainty in illness in cardiac rehabilitation adherence remains unclear, clinicians may have difficulty identifying patients whose poor adherence is driven primarily by cognitive confusion, negative threat appraisal, or emotional avoidance. As a result, interventions may remain overly general, focusing mainly on information provision or exercise prescription without addressing the specific psychological barriers that prevent patients from initiating and sustaining rehabilitation behaviors. More importantly, uncertainty in illness may influence adherence through both cognitive and emotional pathways. Therefore, examining the mechanisms linking uncertainty in illness to cardiac rehabilitation adherence is necessary for translating psychological assessment into precise, tiered, and clinically actionable intervention plans.

Cardiac self-efficacy refers to an individual's belief in and confidence regarding their ability to successfully perform behaviors necessary to maintain cardiac health (20). According to social cognitive theory (21), self-efficacy is a core cognitive determinant of behavioral initiation, persistence, and effort, and profoundly shapes individuals' appraisal of their own condition. Specifically, when patients experience uncertainty regarding their illness, their assessment of their physical condition and functional capacity often becomes more negative and ambiguous (13), making it difficult for them to form clear behavioral expectations and outcome expectations. As a result, their confidence in their ability to carry out rehabilitation behaviors may decline. In this sense, uncertainty in illness may disrupt an individual's cognitive framework, hindering the effective integration of illness-related information and the formation of positive evaluations of one's coping capabilities (22). For example, Wang and Zhang (23) reported a significant negative association between uncertainty in illness and self-efficacy. Although previous studies have recognized the relevance of cardiac self-efficacy to rehabilitation behaviors in patients with CHD (24), few have examined whether uncertainty in illness significantly affects cardiac self-efficacy in this population, and even fewer have tested the mediating role of cardiac self-efficacy in the relationship between uncertainty in illness and cardiac rehabilitation adherence.

Fear of activity refers to an excessive fear arising from concerns that exercise or physical activity may exacerbate pain or cause bodily injury (25). Previous research has shown that, in patients with CHD, fear of activity is primarily manifested as persistent concern and fear that exercise may trigger angina, recurrent myocardial infarction, or even sudden cardiac death (26, 27). Such fears often lead patients to adopt a generalized avoidance strategy toward exercise and physical activity (28). Consequently, fear of activity has gradually emerged as a major barrier to participation in and adherence to exercise-based cardiac rehabilitation among patients with CHD (29, 30). Further analysis suggests that cardiac self-efficacy may be associated with fear of activity (31). When patients lack confidence in their ability to control cardiac symptoms and to engage in exercise safely, they are more likely to interpret physical discomfort experienced during exercise in a catastrophic manner, thereby triggering or exacerbating fear of activity. In other words, insufficient self-efficacy may heighten the sensitivity of individuals' internal threat appraisal system, amplify their subjective perception of exercise-related risks, and ultimately foster fear of activity. Therefore, this study proposes that uncertainty in illness in patients with CHD may first reduce cardiac self-efficacy, which in turn increases fear of activity, ultimately leading to poorer cardiac rehabilitation adherence. This proposition provides a more comprehensive theoretical framework for understanding the internal mechanisms through which uncertainty in illness affects cardiac rehabilitation adherence.

From an integrated self-regulation and fear-avoidance perspective, illness uncertainty may first undermine patients' confidence in managing their cardiac condition and participating safely in rehabilitation, thereby reducing cardiac self-efficacy. Lower cardiac self-efficacy may then increase patients' fear of activity, because individuals who feel less capable of managing symptoms or coping with physical exertion are more likely to interpret bodily sensations during exercise as threatening. This fear may subsequently lead to avoidance of rehabilitation activities and poorer cardiac rehabilitation adherence. Accordingly, cardiac self-efficacy and fear of activity may operate as sequential psychological mechanisms linking illness uncertainty to rehabilitation adherence.

The above theoretical reasoning primarily reflects a variable-centered perspective, which is useful for clarifying the average associations and mediating mechanisms among uncertainty in illness, cardiac self-efficacy, fear of activity, and cardiac rehabilitation adherence. Nevertheless, a variable-centered approach assumes that the estimated relationships are generally applicable to the whole sample and therefore may overlook meaningful heterogeneity among patients. In clinical practice, patients with CHD may not only differ in the overall level of uncertainty in illness, but also show different configurations across its specific dimensions, such as ambiguity, lack of clarity, and unpredictability. As a person-centered method, latent profile analysis can identify latent subgroups with distinct patterns of uncertainty in illness based on individuals' responses across different dimensions, thereby uncovering internal population structures that may be obscured by variable-centered methods. Although latent profile analysis has been increasingly applied in psychological and health behavior research (32, 33), its use in the study of uncertainty in illness among patients with CHD remains largely unexplored. More importantly, if patients belonging to different latent subgroups of uncertainty in illness differ significantly in key variables such as cardiac self-efficacy, fear of activity, and cardiac rehabilitation adherence, this would provide direct evidence to support precise assessment and stratified intervention in clinical practice.

**Figure 1:**
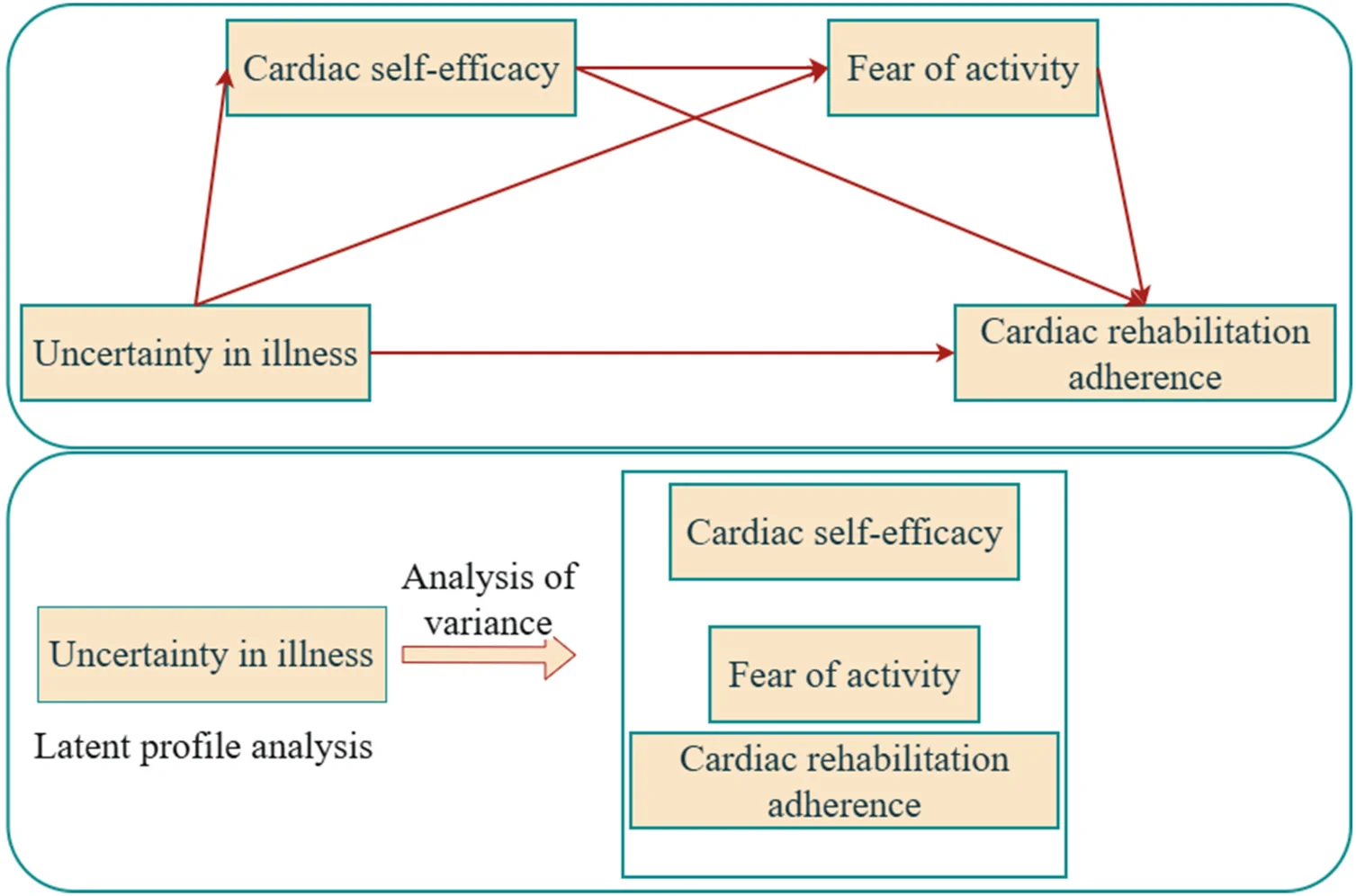
Theoretical framework model.

To address these research gaps, the present cross-sectional study adopts a dual analytical strategy that combines variable-centered and person-centered approaches to systematically investigate the relationship between uncertainty in illness and cardiac rehabilitation adherence. At the variable-centered level, this study aims to identify latent subgroups of uncertainty in illness among patients with CHD through latent profile analysis and further compare differences among these subgroups in cardiac self-efficacy, fear of activity, and cardiac rehabilitation adherence. At the person-centered level, this study aimed to identify latent subgroups of uncertainty in illness among patients with CHD through latent profile analysis and to compare differences among these subgroups in cardiac self-efficacy, fear of activity, and cardiac rehabilitation adherence. The integration of these two analytical approaches may provide complementary evidence regarding both average association patterns and population heterogeneity, thereby offering preliminary evidence for assessment-informed and stratified rehabilitation support, as shown in Figure 1.

Based on the above analysis, this study puts forward the following hypotheses:.

**H1:** Uncertainty in illness is significantly and negatively associated with cardiac rehabilitation adherence in patients with CHD.
**H2:** Cardiac self-efficacy is statistically involved in the indirect association between uncertainty in illness and cardiac rehabilitation adherence in patients with CHD.
**H3**: Fear of activity is statistically involved in the indirect association between uncertainty in illness and cardiac rehabilitation adherence in patients with CHD.
**H4:** Cardiac self-efficacy and fear of activity are sequentially involved in the indirect association between uncertainty in illness and cardiac rehabilitation adherence.
**RQ1:** What latent profiles of uncertainty in illness can be identified among patients with CHD?
**RQ2:** How do demographic and clinical characteristics differ across the identified latent profiles?

## 2 Methods

### 2.1 Study design

This study employed a cross-sectional survey design to systematically examine the relationship between uncertainty in illness and cardiac rehabilitation adherence in patients with coronary heart disease (CHD), as well as the underlying psychological mechanisms. The study protocol was reviewed and approved by the Ethics Committee of First Hospital of Shanxi Medical University (No.:KYLL-2024–159).

Using a convenience sampling strategy, data were collected from August 2024 to April 2025 in the departments of cardiology and cardiac rehabilitation centers of three hospitals in Shanxi Province, China. Before the initiation of data collection, the research team reached a consensus with the heads of the collaborating cardiology departments and cardiac rehabilitation centers regarding the study objectives, eligibility criteria, data collection procedures, and quality control requirements. Potentially eligible patients with CHD were initially screened by the researchers in cardiology wards and outpatient cardiac rehabilitation clinics according to the predefined inclusion and exclusion criteria. For those who met the preliminary screening criteria, the researchers explained the study purpose, content, and confidentiality measures in detail through face-to-face communication when the patients were clinically stable and had sufficient time to participate. Written informed consent was then obtained from each participant. Subsequently, participants were provided with either a paper-based questionnaire or an electronic questionnaire accessed via a QR code, and necessary instructions for questionnaire completion were given on site. After completion, the researchers checked each questionnaire item by item for completeness. Any missing responses were immediately brought to the participant's attention and completed on the spot.

Minimum sample size analysis. The sample size was determined to satisfy the requirements of the two principal analytic approaches used in this study, namely LPA and chain mediation analysis. Based on regression mediation analysis, this study used G*power 3.1 software to evaluate the minimum sample size. This study chose the “*F*-test”, and the statistical test was “Linear multiple regression: Fixed model, R^2^ deviation from zero”, parameter set to effect size f^2^ 0.15, *α* set to 0.05, power set to 0.95 and predictor variable set to 14. The results indicated a minimum sample size of 194 participants. In terms of latent profile analysis, the results of large-scale Monte Carlo simulation based on Tein, Coxe (34) show that when the expected number of profiles is 3–5, the proportion of each profile is not less than 5% and the separation between profiles is moderate, a sample size of at least 400 is usually required to obtain stable and reproducible classification results. Considering the presence of invalid samples during the collection process, the minimum sample size was set to 450 participants in this study.

To enhance the rigor of the study, strict inclusion and exclusion criteria were established. The inclusion criteria were as follows: (1) age ≥18 years; (2) diagnosis of CHD confirmed by coronary angiography; (3) referral by a clinician to a cardiac rehabilitation program or current participation in cardiac rehabilitation; (4) clear consciousness and basic ability to communicate and understand the survey content; and (5) voluntary participation with written informed consent. Because the target population included both patients who had been referred to cardiac rehabilitation and those who were already participating in cardiac rehabilitation, adherence was assessed as a self-reported multidimensional construct reflecting patients' willingness, readiness, and perceived implementation of recommended rehabilitation behaviors. Thus, the outcome did not represent objective attendance at rehabilitation sessions or completion of a standardized rehabilitation program, but rather the patient-reported degree to which individuals intended to follow or perceived themselves as following core cardiac rehabilitation recommendations.

The exclusion criteria were as follows: (1) severe cognitive impairment or psychiatric disorders preventing comprehension of the questionnaire; (2) severe uncontrolled heart failure, malignant arrhythmia, or other life-threatening acute cardiovascular events with unstable clinical status; and (3) severe osteoarticular disease, neuromuscular disease, or other physical dysfunctions that could interfere with study participation. Severe cognitive impairment was defined as an MMSE score below 10 or a documented clinical diagnosis of severe cognitive impairment that prevented participants from understanding the study procedures and completing the questionnaire independently or with assistance.

A total of 512 patients with CHD were approached. Of these, 23 had not yet entered the cardiac rehabilitation stage. During data cleaning, 17 questionnaires were excluded because of highly regular response patterns or excessively short completion times (<2 min). Consequently, 472 valid questionnaires were included in the final analysis, yielding an effective response rate of 92.19%. Among the participants, 295 were male (62.50%) and 177 were female (37.50%). Detailed demographic characteristics are presented in Table 1.

**Table 1:** Summary of participants' demographic characteristics.

Variables	Items	Number (*N*)	Proportion (%)
Gender	Male	295	62.5%
	Female	177	37.5%
Educational background	Primary school and below	126	26.7%
	Middle school—High school	233	49.4%
	Bachelor degree or above	113	23.9%
Place of residence	City	275	58.3%
	Rural area	197	41.7%
Marital status	Married	366	77.5%
	Unmarried	32	6.8%
	Divorced	41	8.7%
	Widowed	33	7.0%
Monthly income level	3,000 RMB and below	73	15.5%
	3,001–6,000 RMB	134	28.4%
	6,001–9,000 RMB	89	18.9%
	9,001 RMB and above	176	37.3%
Types of coronary artery disease	Acute Coronary Syndrome	241	51.1%
	Chronic Coronary Syndrome	231	48.9%
Time to Diagnosis	＜6 months	148	31.40%
	6 months to 1 year	112	23.70%
	1 year and above	212	44.9%
Smoking	Yes.	363	76.9%
	No.	109	23.1%
Drinking alcohol	Yes.	372	78.8%
	No.	100	21.2%
Age (years)		63.17 ± 12.544	

### 2.2 Measures tools

#### 2.2.1 Uncertainty in illness scale

Uncertainty in illness was assessed using the Mishel Uncertainty in Illness Scale originally developed by Mishel (35) and translated into Chinese by Ye, She (36), who further established its cultural applicability and reliability in Chinese patient populations. The scale consists of 20 items covering three dimensions: ambiguity, lack of clarity, and unpredictability. A sample item is: “I do not know how long this illness will last.” In the present study, this scale was used to assess uncertainty in illness among patients with CHD. All items were rated on a 5-point Likert scale ranging from 1 (“strongly disagree”) to 5 (“strongly agree”). Total scores range from 20 to 100, with higher scores indicating greater uncertainty in illness. In this study, Cronbach's alpha for the scale was 0.958.

#### 2.2.2 Cardiac self-efficacy scale

Cardiac self-efficacy was assessed using the Chinese version of the Cardiac Self-Efficacy Scale (CSES). The original scale was developed by Sullivan, LaCroix (37) and later translated and validated in Chinese patients with CHD by Zhang, Zhan (38). In the original validation of the Chinese version, the item “maintain your sexual relationship with your spouse” was removed because of a high non-response rate among older participants, yielding a 12-item, three-dimensional structure (controlling illness, controlling symptoms, and maintaining function). In the present study, however, we administered the full 13-item version, retaining the spousal-relationship item within the maintaining-function dimension. This item was retained because data were collected through on-site, item-by-item verified administration with immediate completion of any missing responses, a procedure that minimized the non-response problem that had motivated its deletion in the original validation study. The three dimensions therefore comprised controlling illness (items 1–4), controlling symptoms (items 5–8), and maintaining function (items 9–13). A sample item is: “How confident are you that you can make your doctor understand your concerns about your heart?” To maintain a consistent response format across all instruments in the survey battery and to reduce respondent burden, each item was rated on a 5-point Likert scale ranging from 1 (“not at all confident”) to 5 (“completely confident”), rather than the 0–4 metric used in the original and Chinese validation studies. Total scores therefore ranged from 13 to 65, with higher scores indicating greater cardiac self-efficacy. In the present study, Cronbach's alpha for the 13-item scale was 0.747, indicating acceptable internal consistency.

#### 2.2.3 Fear of activity scale

Fear of activity was assessed using the Chinese version of the Fear of Activity Scale translated and validated by Chen, Guo (39) in patients with coronary artery disease. The scale includes 21 items covering four dimensions: fear of exercise, avoidance of injury, body perception, and functional role. A sample item is: “Because of my heart condition, I feel uncomfortable when walking uphill.” In this study, the scale was used to assess fear of activity in patients with CHD, with each item rated on a 5-point Likert scale from 1 (“not at all fearful”) to 5 (“extremely fearful”). Total scores range from 21 to 105, with higher scores reflecting greater fear of activity. In this study, Cronbach's alpha for the scale was 0.834, indicating good internal consistency.

#### 2.2.4 Cardiac rehabilitation adherence

Cardiac rehabilitation adherence was measured using the Chinese version of the Cardiac Rehabilitation Adherence Scale developed by Yao, Wen (40), which has demonstrated satisfactory cultural applicability and reliability in patients with CHD. The scale comprises 33 items across five dimensions: exercise adherence, medication adherence, risk factor management adherence, nutritional management adherence, and psychological management adherence. In the present study, participants rated each item on a 5-point Likert scale ranging from 1 (“very unwilling”) to 5 (“very willing”). Total scores range from 33 to 165, with higher scores indicating better cardiac rehabilitation adherence. In this study, Cronbach's alpha for the scale was 0.903, indicating good internal consistency.

### 2.3 Statistical analysis

All statistical analyses were performed using SPSS 27.0 and Mplus 8.3, with the significance level set at *α* = 0.05. First, routine analyses were conducted, including reliability analysis, common method bias testing, descriptive statistics, correlation analysis, and between-group comparisons.

At the variable-centered level, a theory-driven statistical mediation model was examined using Model 6 of the PROCESS macro. The model examined the direct association between uncertainty in illness and cardiac rehabilitation adherence, as well as the indirect associations involving cardiac self-efficacy and fear of activity. The significance of indirect associations was tested using the bias-corrected percentile Bootstrap method with 5,000 resamples. An indirect association was considered statistically significant if the 95% confidence interval did not include 0. The proportion of each indirect association relative to the total association was calculated to describe the relative contribution of different statistical pathways.

At the person-centered level, latent profile analysis was performed to identify latent subgroups of uncertainty in illness. Scores for each items of uncertainty in illness were used as indicator variables, and models with one to five profiles were fitted sequentially. The optimal number of profiles was determined comprehensively based on the Akaike information criterion (AIC), Bayesian information criterion (BIC), sample-size adjusted BIC (aBIC), Lo-Mendell-Rubin likelihood ratio test (LMR-LRT), bootstrap likelihood ratio test (BLRT), and entropy, with the additional requirements that each profile contain no less than 5% of the total sample and demonstrate clear clinical interpretability.

To address the potential sensitivity of high-dimensional LPA to starting values, all models were estimated with 500 random sets of start values and 100 final-stage optimizations; the best log-likelihood was replicated across multiple starts, indicating that solutions were not local maxima. Models converged normally without boundary or non-positive-definite estimates.

After the optimal profile structure was identified, profile membership was used as the grouping variable, and one-way analysis of variance was conducted to compare differences among profiles in cardiac self-efficacy, fear of activity, and cardiac rehabilitation adherence.

## 3 Results

### 3.1 Common method bias

Given that all study variables were collected through patient self-report, potential common method bias could not be completely ruled out, although anonymous data collection and confidentiality measures were adopted to reduce social desirability bias. Therefore, Harman's single-factor test was conducted by entering all measurement items from all scales into an unrotated exploratory factor analysis. The results showed that the first unrotated common factor accounted for 16.683% of the total variance, which was below the critical threshold of 40%. This result suggested that no single dominant factor accounted for the majority of covariance among the measured variables. However, this result should be interpreted cautiously because the test cannot fully address potential common-method bias arising from same-source and same-time self-report measurement.

### 3.2 Descriptive statistics and correlation analysis

The descriptive statistics and correlations among the core study variables are presented in Table 2. Specifically, the skewness values ranged from −0.126 to −0.012, and the kurtosis values ranged from −0.764 to −0.142. According to the criteria proposed by Kline (41), the distributions of the core variables could be considered approximately normal.

**Table 2:** Descriptive statistics and correlation analysis of the core variables.

Variables	M	SD	Skewness	Kurtosis	1	2	3	4
Uncertainty in illness	3.133	0.949	−0.112	−0.764	1			
Cardiac self-efficacy	3.108	0.573	−0.126	−0.361	−0.162***	1		
Fear of activity	3.114	0.588	−0.028	−0.142	0.270***	−0.339***	1	
Cardiac rehabilitation adherence	2.992	0.624	−0.012	−0.294	−0.308***	0.396***	−0.452***	1

*** *p* *<* *0.001.*

Correlation analysis showed that uncertainty in illness was significantly and negatively correlated with cardiac self-efficacy (*r* = −0.162, *p* < 0.001), significantly and positively correlated with fear of activity (*r* = 0.270, *p* < 0.001), and significantly and negatively correlated with cardiac rehabilitation adherence (*r* = −0.308, *p* < 0.001). Cardiac self-efficacy was significantly and negatively correlated with fear of activity (*r* = −0.339, *p* < 0.001) and significantly and positively correlated with cardiac rehabilitation adherence (*r* = 0.396, *p* < 0.001). Fear of activity was significantly and negatively correlated with cardiac rehabilitation adherence (*r* = −0.452, *p* < 0.001).

### 3.3 Analysis between groups

To further explore the differences of demographic information in compliance with cardiac rehabilitation, this study used independent sample *t*-test for dichotomous variables and one-way ANOVA for multivariate variables for comparison between groups, as shown in the Table 3. The results showed that gender, education background, residence, marital status, monthly income level, type of coronary artery disease, time of disease diagnosis, smoking and drinking had no significant difference in compliance with cardiac rehabilitation (*p* > 0.05).

**Table 3:** Group analysis of demographic information on adherence to cardiac rehabilitation.

Variables	Items	M	SD	*t*/F	*p*	Cohen’s d/*η*^2^
Gender	Male	2.973	0.619	0.860	0.390	0.082
	Female	3.024	0.633			
Educational background	Primary school and below	3.010	0.632	0.274	0.761	0.001
	Middle school—High school	3.001	0.620			
	Bachelor degree or above	2.955	0.627			
Place of residence	Rural area	2.998	0.560	0.179	0.858	0.017
	City	2.988	0.667			
Marital status	Divorced	3.188	0.619	2.220	0.085	0.014
	Widowed	2.820	0.590			
	Unmarried	2.967	0.572			
	Married	2.988	0.629			
Monthly income level	3,000 RMB and below	3.061	0.646	1.375	0.250	0.009
	3,001–6,000 RMB	2.986	0.618			
	6,001–9,000 RMB	3.071	0.622			
	9,001 RMB and above	2.929	0.619			
Types of coronary artery disease	Chronic Coronary Syndrome	2.966	0.602	−0.884	0.377	−0.081
	Acute Coronary Syndrome	3.017	0.645			
Time to Diagnosis	＜6 months	3.008	0.646	2.758	0.064	0.012
	6 months to 1 year	2.875	0.633			
	1 year and above	3.043	0.598			
Smoking	Yes.	2.895	0.598			
	No.	3.021	0.629			
Drinking alcohol	Yes.	3.047	0.661	0.988	0.324	0.111
	No.	2.978	0.614			

### 3.4 Statistical indirect association analysis involving cardiac self-efficacy and fear of activity

In this study, PROCESS macro Model 6 was used to examine a theory-driven statistical indirect association model, with uncertainty in illness as the independent variable, cardiac rehabilitation adherence as the dependent variable, and cardiac self-efficacy and fear of activity as intervening variables. At the same time, in view of the correlation analysis between demographic information and core study variables, no significant correlation was found between demographic information and core study variables. More importantly, the between-group analysis showed that demographic information did not significantly differ in adherence to cardiac rehabilitation. Based on this, demographic information was not included in the main model as a control variable in this study.

**Figure 2:**
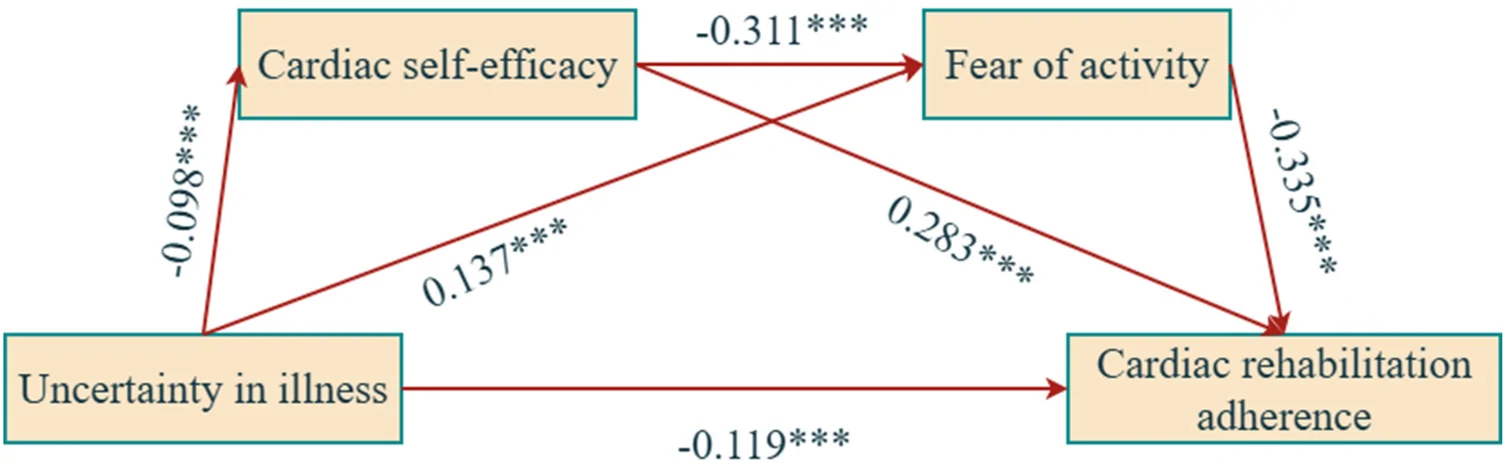
Path coefficients of the chain mediation model. ***p < 0.001.

The regression results are shown in Table 4 and Figure 2. Uncertainty in illness was significantly and negatively associated with cardiac self-efficacy (B = −0.098, *p* < 0.001). Uncertainty in illness was significantly and positively associated with fear of activity (B = 0.137, *p* < 0.001), while cardiac self-efficacy was significantly and negatively associated with fear of activity (B = −0.311, *p* < 0.001). In addition, uncertainty in illness was significantly and negatively associated with cardiac rehabilitation adherence (B = −0.119, *p* < 0.001); cardiac self-efficacy was significantly and positively associated with cardiac rehabilitation adherence (B = 0.283, *p* < 0.001); and fear of activity was significantly and negatively associated with cardiac rehabilitation adherence (B = −0.335, *p* < 0.001).

**Table 4:** Conditional associations among uncertainty in illness, cardiac self-efficacy, fear of activity, and cardiac rehabilitation adherence.

Predictive variable	Equation 1: Cardiac self-efficacy			Equation 2: Fear of activity			Equation 3: Cardiac rehabilitation adherence		
	*B*	*SE*	*t*	*B*	*SE*	*t*	*B*	*SE*	*t*
Uncertainty in illness	−0.098	0.028	−3.561***	0.137	0.027	5.150***	−0.119	0.027	−4.495***
Cardiac self-efficacy				−0.311	0.044	−7.080***	0.283	0.045	6.296***
Fear of activity							−0.335	0.045	−7.459***
*R^2^*	0.026			0.162			0.301		
*F*	12.677***			45.427***			67.086***		

*** *p* *<* *0.001.*

For the statistical indirect association analysis, the bias-corrected percentile Bootstrap method with 5,000 resamples was used to test the significance of the total, direct, and indirect associations, as shown in Table 5. The results indicated that the total association between uncertainty in illness and cardiac rehabilitation adherence was −0.203 [95% CI = (−0.259, −0.146)], supporting Hypothesis 1. The direct association between uncertainty in illness and cardiac rehabilitation adherence was −0.119 [95% CI = (−0.171, −0.067)]; because the confidence interval did not include 0, the direct association was significant and accounted for 59% of the total association. The total indirect association through cardiac self-efficacy and fear of activity was −0.084 [95% CI = (−0.115, −0.054)], accounting for 41% of the total effect.

**Table 5:** Decomposition of total, direct, and indirect statistical associations.

Effect type	Effect	SE	LLCI	ULCL	Effect proportion	Supported hypothesis
Total effect	−0.203	0.029	−0.259	−0.146		
Direct effect	−0.119	0.027	−0.171	−0.067	59%	H1
Indirect effect	−0.084	0.016	−0.115	−0.054	41%	
Ind 1	−0.028	0.009	−0.046	−0.012	14%	H2
Ind 2	−0.046	0.011	−0.068	−0.026	23%	H3
Ind 3	−0.010	0.004	−0.018	−0.004	5%	H4

Ind 1: Uncertainty in illness → Cardiac self-efficacy → Cardiac rehabilitation adherence; Ind 2: Uncertainty in illness → Fear of activity → Cardiac rehabilitation adherence; Ind 3: Uncertainty in illness → Cardiac self-efficacy → Fear of activity → Cardiac rehabilitation adherence.

Specifically, the indirect association for the pathway uncertainty in illness → cardiac self-efficacy → cardiac rehabilitation adherence was −0.028 [95% CI = (−0.046, −0.012)], accounting for 14% of the total association and supporting Hypothesis 2. The indirect association for the pathway uncertainty in illness → fear of activity → cardiac rehabilitation adherence was −0.046 [95% CI = (−0.068, −0.026)], supporting Hypothesis 3. The sequential indirect association for the pathway uncertainty in illness →  self-efficacy → fear of activity → cardiac rehabilitation adherence was −0.010 [95% CI = (−0.018, −0.004)], accounting for 5% of the total effect and supporting Hypothesis 4. These findings suggest that uncertainty in illness may reduce cardiac rehabilitation adherence by weakening patients' cardiac self-efficacy; patients with insufficient self-efficacy may then be more likely to interpret bodily discomfort during exercise situations in a catastrophic and threatening manner, thereby triggering or exacerbating fear of activity and ultimately lowering adherence to cardiac rehabilitation.

Test of robustness. To further enhance the accuracy and reliability of the statistical indirect association of cardiac self-efficacy and fear of exercise, we included age, gender, education, and disease duration as control variables in the model. The results of the study showed that the sequential indirect association involving cardiac self-efficacy and exercise fear still had a significant effect [Effect = −0.011, SE = 0.004, 95%CI = (−0.019, −0.004)], and the difference was small compared with the effect size of the chain mediation model without control variables, as shown in Table 6.

**Table 6:** Decomposition of the chain mediation effect (including age, gender, education and disease duration).

Effect type	Effect	SE	LLCI	ULCL	Effect proportion	Supported hypothesis
Total effect	−0.202	0.029	−0.259	−0.146	100.0%	
Direct effect	−0.117	0.027	−0.169	−0.065	57.8%	H1
Indirect effect	−0.085	0.015	−0.117	−0.056	42.2%	
Ind1	−0.029	0.009	−0.047	−0.013	14.2%	H2
Ind2	−0.046	0.011	−0.068	−0.025	22.7%	H3
Ind3	−0.011	0.004	−0.019	−0.004	5.2%	H4

Ind 1: Uncertainty in illness → Cardiac self-efficacy → Cardiac rehabilitation adherence; Ind 2: Uncertainty in illness → Fear of activity → Cardiac rehabilitation adherence; Ind 3: Uncertainty in illness → Cardiac self-efficacy → Fear of activity → Cardiac rehabilitation adherence.

### 3.5 Latent profile analysis of uncertainty in illness

To identify the latent subgroup structure of uncertainty in illness among patients with CHD, latent profile models with one to five classes were sequentially fitted using the scores of the 20 items of the uncertainty in illness scale as indicator variables. The model fit indices are presented in Tables 7–9.

**Table 7:** Fit indices for the 1- to 5-class latent profile models (20 items).

Indices	Profile 1	Profile 2	**Profile 3**	Profile 4	Profile 5
AIC	31,390.977	27,392.908	**26,364**.**189**	25,982.240	25,822.908
BIC	31,557.256	27,646.484	**26,705**.**062**	26,410.408	26,338.373
aBIC	31,430.303	27,452.880	**26,444**.**808**	26,083.504	25,944.819
Entropy	–	0.956	**0**.**930**	0.909	0.895
LMR (p)	–	< 0.001	**<0** **.** **001**	0.264	0.201
BLRT (p)	–	< 0.001	**<0** **.** **001**	<0.001	<0.001
Class 1	–	46.28%	**26**.**27%**	15.89%	13.14%
Class 2	–	53.72%	**39**.**41%**	28.81%	26.69%
Class 3	–	–	**34**.**32%**	31.36%	24.79%
Class 4	–	–	–	23.94%	25.00%
Class 5	–	–	–	–	10.38%

AIC, Akaike information criterion; BIC, Bayesian information criterion; aBIC, sample-size adjusted Bayesian information criterion; LRT, Lo-Mendell-Rubin likelihood ratio test; BLRT, bootstrap likelihood ratio test. Class proportions are based on most likely class membership.

**Table 8:** Classification diagnostics for the optimal three-profile model.

Classification indicator	Low	Moderate	High
Estimated model-based count	120.96	188.3	162.74
Estimated model-based proportion	25.63%	39.89%	34.48%
Most likely class count	124	186	162
Most likely class proportion	26.27%	39.41%	34.32%
Average posterior probability	0.959	0.963	0.975

Low, Cognitively clear–low uncertainty group; Medium, Partially informed–moderate uncertainty group; High, Cognitively ambiguous–high uncertainty group.

**Table 9:** Average posterior probability.

Most likely assigned profile	Probability of Profile 1	Probability of Profile 2	Probability of Profile 3
Profile 1	0.959	0.041	0.000
Profile 2	0.011	0.963	0.026
Profile 3	0.000	0.025	0.975

With regard to the information criteria, the AIC, BIC, and aBIC all decreased continuously as the number of latent classes increased. In addition, the Lo-Mendell-Rubin adjusted likelihood ratio test for the three-class model was significant, indicating that the three-class model fit the data significantly better than the two-class model. The entropy values of all multi-class models were above the acceptable threshold of 0.80, suggesting adequate classification accuracy. Finally, in the three-class model, the proportions of the three latent classes were 26.27%, 39.41%, and 34.32%, respectively, all well above the minimum requirement of 5% of the total sample, indicating sufficient sample size for each class. Therefore, the three-class latent profile model was determined to be the optimal model.

**Figure 3:**
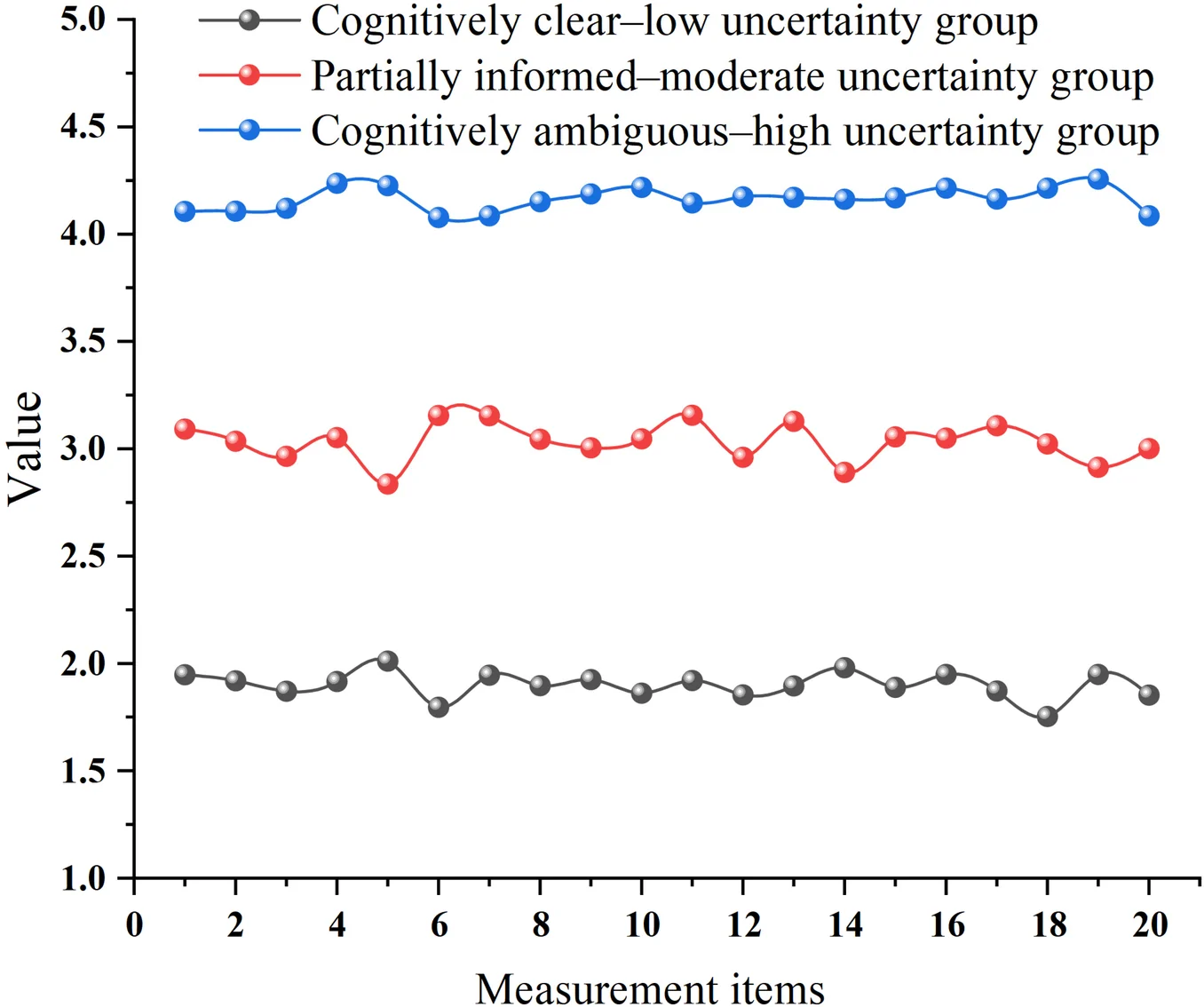
Profile plot of the optimal latent profiles.

Based on the above results, the conditional means of the three-class latent profile model across all measurement items of uncertainty in illness are shown in Figure 3. According to the score levels across the three dimensions and the overall response patterns, the three latent profiles were labeled as follows. Class 1 was labeled the cognitively clear–low uncertainty group (*n* = 124, 26.27%), characterized by relatively clear understanding of disease-related information, lower ambiguity regarding symptoms, less perceived lack of information, and lower unpredictability regarding disease progression and prognosis. Class 2 was labeled the partially informed–moderate uncertainty group (*n* = 186, 39.41%), representing the most common pattern of uncertainty in illness among patients with CHD. Class 3 was labeled the cognitively ambiguous–high uncertainty group (*n* = 162, 34.32%), characterized by high levels of ambiguity, lack of information, and unpredictability regarding the disease, indicating the most prominent and intense experience of uncertainty in illness. These three distinct latent profile classes demonstrated clear heterogeneity in uncertainty in illness among patients with CHD, thereby supporting RQ1.

Test of robustness. In order to further enhance the accuracy of profile analysis, taking the scores of the three dimensions of the Mishel Uncertainty in Illness Scale as the index variables, 1–5 types of latent profile models were fitted sequentially to determine the potential subgroup structure of the uncertainty in illness of patients with coronary heart disease. The model fit index is shown in Table 10. The results showed that, with regard to the information criteria, the AIC, BIC, and aBIC all decreased continuously as the number of latent classes increased. In addition, the Lo-Mendell-Rubin adjusted likelihood ratio test for the three-class model was significant, indicating that the three-class model fit the data significantly better than the two-class model. The three classes of profiles were again verified to be the optimal profile types.

**Table 10:** Fit indices for the 1- to 5-class latent profile models (three dimensions).

Indices	Profile 1	Profile 2	**Profile 3**	Profile 4	Profile 5
AIC	3,999.893	3,164.856	**2,815**.**850**	2,627.876	2,531.493
BIC	4,024.835	3,206.426	**2,874**.**048**	2,702.702	2,622.946
aBIC	4,005.792	3,174.688	**2,829**.**614**	2,645.573	2,553.122
Entropy	–	0.879	**0**.**873**	0.865	0.860
LMR (p)	–	<0.001	**<0** **.** **001**	0.256	0.018
BLRT (p)	–	<0.001	**<0** **.** **001**	<0.001	<0.001
Class 1	–	46.61%	**24**.**58%**	16.95%	13.56%
Class 2	–	53.39%	**24**.**11%**	29.66%	25.64%
Class 3	–	–	**41**.**31%**	19.07%	25.42%
Class 4	–	–	–	34.32%	10.59%
Class 5	–	–	–	–	24.79%

AIC, Akaike information criterion; BIC, Bayesian information criterion; aBIC, sample-size adjusted Bayesian information criterion; LRT, Lo-Mendell-Rubin likelihood ratio test; BLRT, bootstrap likelihood ratio test. Class proportions are based on most likely class membership.

Bold values indicates the optimal latent profile.

### 3.6 Differences among the optimal latent profiles in cardiac self-efficacy, fear of activity, and cardiac rehabilitation adherence

**Figure 4:**
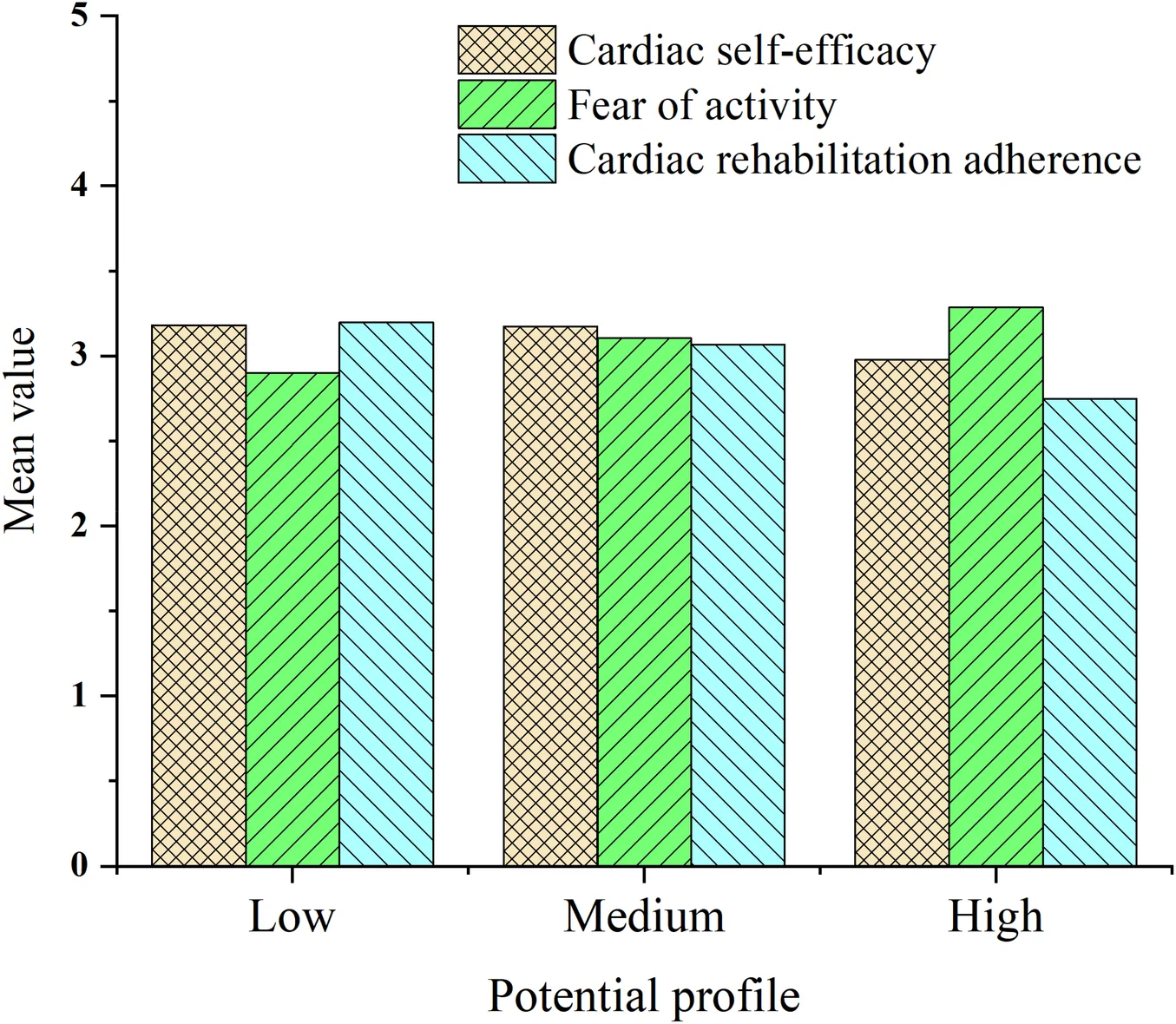
Differences among the optimal latent profiles in cardiac self-efficacy, fear of activity, and cardiac rehabilitation adherence. Low, Cognitively clear–low uncertainty group; Medium = Partially informed–moderate uncertainty group; High, Cognitively ambiguous–high uncertainty group.

To further examine differences in cardiac self-efficacy, fear of activity, and cardiac rehabilitation adherence across the latent classes of uncertainty in illness, one-way analysis of variance was conducted with latent profile membership as the independent variable. The results are shown in Table 11 and Figure 4.

**Table 11:** Analysis of variance of cardiac self-efficacy, fear of activity, and cardiac rehabilitation adherence across the optimal latent profiles.

Latent profiles	Cardiac self-efficacy		Fear of activity		Cardiac rehabilitation adherence	
	M	SD	M	SD	M	SD
Low	3.181	0.556	2.902	0.559	3.197	0.591
Medium	3.173	0.571	3.105	0.567	3.068	0.586
High	2.978	0.569	3.285	0.581	2.749	0.616
p	0.002		<0.001		<0.001	
F	6.513		15.899		22.202	
η2	0.027		0.063		0.086	

Means sharing the same superscript letter do not differ significantly; means with different superscript letters differ significantly after bonferroni correction. Low, Cognitively clear–low uncertainty group; Medium, Partially informed–moderate uncertainty group; High, Cognitively ambiguous–high uncertainty group.

The results showed significant differences among the three latent classes in cardiac self-efficacy (*F* = 6.513, *p* = 0.002, *η*^2^ = 0.027), fear of activity (*F* = 15.899, *p* < 0.001, *η*^2^ = 0.063), and cardiac rehabilitation adherence (F = 22.202, *p* < 0.001, *η*^2^ = 0.086), supporting RQ2. Overall, the cognitively ambiguous–high uncertainty group showed the least favorable performance across all three variables, with the lowest cardiac self-efficacy, the highest fear of activity, and the poorest cardiac rehabilitation adherence. These findings suggest that patients with CHD in the high uncertainty in illness profile should be a key target population for clinical intervention.

Bonferroni-corrected *post-hoc* pairwise comparisons further clarified the pattern of group differences. For cardiac self-efficacy, the cognitively ambiguous–high uncertainty group scored significantly lower than both the cognitively clear–low uncertainty group and partially informed–moderate uncertainty group, whereas the difference between the cognitively clear–low uncertainty group and partially informed–moderate uncertainty group was not significant. For fear of activity, all three pairwise comparisons were significant, indicating a progressive increase from the cognitively clear–low uncertainty group to the partially informed–moderate uncertainty group and then to the cognitively ambiguous–high uncertainty group. For cardiac rehabilitation adherence, the cognitively ambiguous–high uncertainty group reported significantly lower adherence than both the cognitively clear–low uncertainty group and partially informed–moderate uncertainty groups, whereas the difference between the low and partially informed–moderate uncertainty groups was not significant after correction.

## 4 Discussion

### 4.1 Variable-centered analysis

This study confirmed that uncertainty in illness was significantly and negatively associated with cardiac rehabilitation adherence, supporting Hypothesis 1. According to Mishel's Uncertainty in Illness Theory (42), when patients appraise illness-related uncertainty as a threatening stimulus, they are more likely to activate avoidance-oriented coping strategies, which in turn weakens their motivation and sustained engagement in health-promoting behaviors (43). Although previous studies have established a significant negative association between uncertainty in illness and treatment adherence as well as self-management behaviors (44, 45), uncertainty in illness among patients with CHD has distinctive clinical characteristics. On the one hand, although percutaneous coronary intervention or coronary artery bypass grafting effectively restores coronary blood flow during the acute phase (46), patients remain at risk of long-term complications such as in-stent restenosis and graft occlusion after surgery; persistent concern about disease recurrence therefore constitutes an important source of uncertainty in illness. On the other hand, cardiac rehabilitation involves multidimensional and complex intervention programs, and the multiplicity and complexity of such information may further exacerbate patients' cognitive confusion and difficulties in information processing, thereby intensifying their experience of uncertainty in illness. Therefore, the present findings provide direct empirical evidence for understanding the cognitive roots of poor cardiac rehabilitation adherence among patients with CHD in clinical practice.

This study also confirmed the statistically significant indirect association involving cardiac self-efficacy in the relationship between uncertainty in illness and cardiac rehabilitation adherence, supporting Hypothesis 2. According to Bandura's Social Cognitive Theory, the formation of self-efficacy depends heavily on individuals' cognitive appraisal of their own capabilities and environmental conditions (21). When patients with CHD experience high levels of uncertainty in illness, their perceptions of their disease status, treatment effectiveness, and prospects for functional recovery become vague and unstable, making it difficult for them to form clear behavioral expectations and outcome expectations. This cognitively uncertain state undermines the information-processing foundation necessary for the development of self-efficacy, preventing patients from reliably assessing their ability to successfully perform rehabilitation behaviors based on internal information, and thereby leading to reduced cardiac self-efficacy (47). Reduced cardiac self-efficacy further weakens patients' motivation and confidence to initiate and maintain cardiac rehabilitation behaviors, ultimately manifesting as lower rehabilitation adherence. This mediating pathway suggests that uncertainty in illness does not simply reduce rehabilitation adherence directly; rather, it exerts an indirect negative effect by undermining patients' core cognitive resources, namely self-efficacy beliefs. It should be noted, however, that this cognitive pathway may be reciprocal: diminished cardiac self-efficacy could further heighten patients' perception of illness uncertainty, suggesting a mutually reinforcing relationship that warrants confirmation in longitudinal research.

At the same time, this study verified the statistically significant indirect association involving fear of activity in the relationship between uncertainty in illness and cardiac rehabilitation adherence, supporting Hypothesis 3. This finding suggests that among patients with CHD, fear of activity, as an emotional-behavioral response, constitutes an important psychological pathway through which uncertainty in illness affects cardiac rehabilitation adherence. Specifically, high levels of uncertainty in illness make it difficult for patients with CHD to accurately judge the safety boundaries of exercise behavior (48). When patients are unable to determine whether exercise may trigger angina, recurrent myocardial infarction, or even sudden cardiac death, their threat appraisal of exercise situations is substantially amplified, which in turn generates or aggravates fear of activity. Fear of activity may lead patients to broadly avoid exercise and physical activity (25, 49), viewing non-participation in exercise as an adaptive strategy to protect their safety, and ultimately resulting in substantially reduced adherence to exercise-based cardiac rehabilitation. This result is consistent with the predictions of the fear-avoidance model (50), which emphasizes that when individuals establish a conditioned association between physical activity and pain or injury, fear drives the persistence and generalization of avoidance behavior.

More importantly, this study confirmed the statistically significant sequential indirect association involving cardiac self-efficacy and fear of activity in the relationship between uncertainty in illness and cardiac rehabilitation adherence, supporting Hypothesis 4. The statistically significant sequential indirect association reveals a psychological transmission pathway from cognitive appraisal to emotional response and then to behavioral outcomes. Specifically, uncertainty in illness first weakens cardiac self-efficacy in patients with CHD, and patients with insufficient self-efficacy are more likely to generate catastrophic interpretations and threat appraisals when facing exercise situations, thereby triggering or intensifying fear of activity, which ultimately reduces cardiac rehabilitation adherence. From an integrated cognitive-emotional-behavioral perspective (51), uncertainty in illness disrupts patients' positive evaluation of their own capabilities, resulting in lower cardiac self-efficacy; low cardiac self-efficacy then heightens the sensitivity of patients' internal threat appraisal systems and amplifies subjective perceptions of exercise-related risk, thereby giving rise to fear of activity; finally, fear of activity, as a strong negative emotional driver, suppresses patients' participation in and persistence with cardiac rehabilitation (52). This finding provides a more refined theoretical explanation of the internal process through which uncertainty in illness influences cardiac rehabilitation adherence.

Notably, although self-efficacy and fear of activity together mediated 41% of the association between illness uncertainty and rehabilitation adherence, the direct effect still accounted for 59% of the total effect. This indicates that additional mediating or moderating mechanisms are likely to operate within this pathway. Several candidates warrant attention in future research. Social support may buffer the adverse impact of illness uncertainty by providing informational and emotional resources that sustain engagement in rehabilitation (53). Health literacy could shape how patients interpret ambiguous illness-related information (54), thereby influencing both the experience of uncertainty and the capacity to act on rehabilitation recommendations. Coping strategies, particularly the balance between problem-focused and avoidant coping, may determine whether uncertainty translates into disengagement or adaptive self-management (55). Finally, patient–provider communication may attenuate uncertainty directly by clarifying prognosis and treatment expectations (56), while also strengthening patients' confidence in their rehabilitation plan. Incorporating these variables into more comprehensive models would help to explain the substantial direct effect observed here and to identify additional intervention targets for enhancing adherence.

### 4.2 Person-centered analysis

Using latent profile analysis, this study identified three latent subgroups of uncertainty in illness among patients with CHD: a cognitively clear–low uncertainty group (26.27%), a partially informed–moderate uncertainty group (39.41%), and a cognitively ambiguous–high uncertainty group (34.32%), thereby supporting RQ1. The partially informed–moderate uncertainty group accounted for the largest proportion and represented the most common pattern of uncertainty in illness among patients with CHD. This intermediate state may be related to the characteristics of CHD treatment itself: although percutaneous coronary intervention or coronary artery bypass grafting can alleviate acute symptoms and anxiety to some extent, the complexity of long-term postoperative management and the potential risk of disease recurrence prevent patients from completely eliminating uncertainty. Patients in the cognitively ambiguous–high uncertainty group showed pronounced ambiguity, lack of information, and unpredictability regarding their disease status. These patients may have particular difficulties such as limited access to information, low health literacy, or insufficient physician-patient communication, making it difficult for them to effectively integrate and understand complex information related to the disease and rehabilitation. By contrast, patients in the cognitively clear–low uncertainty group were able to form a relatively clear and stable cognitive framework regarding their disease after surgery, which may be associated with higher health literacy, stronger social support networks, or more adequate communication with healthcare providers.

The partially informed–moderate uncertainty group constituted the largest subgroup (39.41%) and therefore represents the most prevalent and clinically representative phenotype among patients with CHD. Clinically, these patients appear to occupy a transitional and potentially unstable cognitive position: they have acquired partial understanding of their disease and treatment—often sufficient to alleviate acute distress—yet retain residual ambiguity regarding long-term prognosis, recurrence risk, and the safety boundaries of rehabilitation activity. Their intermediate scores on cardiac self-efficacy and fear of activity suggest that their illness appraisal has not yet consolidated into either an adaptive or a maladaptive pattern. This intermediate state may render them particularly susceptible to fluctuation: positive experiences and adequate information may move them toward the low uncertainty pattern, whereas adverse events, symptom recurrence, or conflicting information may push them toward the high uncertainty pattern. Because of their large numbers and modifiable cognitive status, this subgroup may represent a high-yield target for preventive intervention aimed at averting progression toward high uncertainty.

Further analyses found significant differences among the latent subgroups of uncertainty in illness in cardiac self-efficacy, fear of activity, and cardiac rehabilitation adherence, thereby supporting RQ2. Specifically, the cognitively ambiguous–high uncertainty group showed the least favorable outcomes across all three variables, with the lowest cardiac self-efficacy, the highest fear of activity, and the poorest cardiac rehabilitation adherence. From a person-centered perspective, this finding provides strong complementary evidence for the mediating mechanisms identified at the variable-centered level. Variable-centered analysis reveals general patterns of association among variables, whereas person-centered analysis further demonstrates that the strength of these general patterns varies significantly across patient subgroups, with patients in the high uncertainty in illness category showing the most pronounced negative psychological mechanisms. In addition, regarding the effect sizes for fear of activity and cardiac rehabilitation adherence, latent class membership explained more variance in these two variables than in cardiac self-efficacy. This finding suggests that subgroup differences in uncertainty in illness are expressed more prominently at the emotional and behavioral levels than at the cognitive level.

### 4.3 Practical and clinical implications

The findings of this cross-sectional study may provide preliminary clinical implications for assessment and hypothesis generation. At the cognitive source, clinicians should place greater emphasis on reducing uncertainty in illness among patients with CHD by providing structured and personalized disease-related education to enhance patients' understanding of disease symptoms, treatment regimens, and rehabilitation processes. For example, evidence-based information support programs could be developed for patients with CHD, covering postoperative recovery timelines, normalization of common bodily responses, simplified explanations of medication mechanisms, and expected goals at different stages of cardiac rehabilitation, thereby systematically reducing patients' cognitive ambiguity and unpredictability regarding illness-related events.

Along the mediating pathway, clinical interventions should simultaneously focus on enhancing cardiac self-efficacy and alleviating fear of activity. To improve cardiac self-efficacy, the four major sources of self-efficacy proposed in Social Cognitive Theory may be applied: graded exercise experiences can provide mastery experiences, peer role models can offer vicarious experiences, positive feedback from the cardiac rehabilitation team can serve as verbal persuasion, and relaxation training and psychological support can reduce physiological arousal. To alleviate fear of activity, exposure-based approaches and cognitive restructuring techniques derived from cognitive behavioral therapy may be adopted to help patients gradually increase exercise intensity and duration in a safely monitored environment, thereby correcting catastrophic beliefs about exercise-related risk through repeated safe exercise experiences.

The three latent subgroups of uncertainty in illness identified in this study also provide direct evidence for precision assessment and stratified intervention in clinical practice. We suggest that systematic screening for uncertainty in illness be incorporated into the initial assessment stage of cardiac rehabilitation so that patients can be categorized into low, moderate, and cognitively ambiguous–high uncertainty groups and receive differentiated intervention strategies accordingly. For patients in the cognitively ambiguous–high uncertainty group, because they showed the poorest performance in cardiac self-efficacy, fear of activity, and cardiac rehabilitation adherence, clinical intervention should prioritize them as a key target population and provide more intensive and comprehensive intervention programs, including in-depth disease information education, systematic self-efficacy enhancement training, professional cognitive-behavioral intervention for fear of activity, and more frequent follow-up and support. For patients in the cognitively clear–low uncertainty group, routine health education and regular follow-up may be sufficient, although continuous monitoring of psychological status remains necessary to prevent increases in uncertainty during rehabilitation due to unexpected events or disease fluctuations.

For the partially informed–moderate uncertainty group, given its large size and cognitively transitional nature, interventions should focus on consolidating existing understanding and preventing escalation of uncertainty rather than on intensive remediation. First, a brief individualized assessment could identify which specific dimension of uncertainty remains most prominent for each patient—ambiguity about symptoms, gaps in disease-related information, or unpredictability of prognosis—so that targeted clarification can be provided. Second, these patients may benefit particularly from structured information reinforcement at key transition points (e.g., discharge, the shift from supervised to home-based rehabilitation, and follow-up visits), when uncertainty is most likely to resurface. Third, low-intensity self-efficacy support—such as goal-setting, self-monitoring of rehabilitation progress, and timely positive feedback—may help stabilize their confidence before fear of activity becomes entrenched. Finally, periodic re-screening of uncertainty is advisable, because their intermediate and potentially unstable status means that early detection of any upward drift would allow timely escalation of support, thereby preventing transition into the high uncertainty group.

The three latent profiles identified here can serve as a triage framework for stratified, cost-sensitive resource allocation. For the cognitively clear–low uncertainty group, who already possess a relatively stable illness cognition and the most favorable self-efficacy, fear-of-activity, and adherence profiles, routine, low-intensity management—standardized health education, group-based guidance, and scheduled follow-up—may be sufficient. This approach minimizes the consumption of specialist resources for patients least likely to need them. For the partially informed–moderate uncertainty group, intermediate-intensity and largely nurse-led or protocol-driven interventions (e.g., targeted information reinforcement and brief self-efficacy support at key transition points) may achieve adequate benefit without requiring the full multidisciplinary apparatus. The most resource-intensive components—coordinated input from cardiologists, rehabilitation therapists, and clinical psychologists, together with cognitive-behavioral intervention for fear of activity and more frequent follow-up—should be preferentially concentrated on the cognitively ambiguous–high uncertainty group, who demonstrated the greatest psychological burden and the poorest adherence and who are therefore most likely to derive incremental benefit from intensive, specialist-delivered care. Such a profile-guided, tiered allocation model reframes multidisciplinary collaboration from an all-or-nothing standard into a scalable resource that can be deployed in proportion to need. For primary hospitals with limited personnel and funding, this offers a pragmatic pathway: rather than attempting to deliver comprehensive multidisciplinary care to every patient—which is often infeasible—institutions can implement brief uncertainty screening at intake and reserve their scarce specialist resources for the high uncertainty subgroup, while managing lower-risk patients through standardized, lower-cost pathways. The heterogeneity identified in this study can be translated into a concrete strategy for improving both the efficiency and the equity of cardiac rehabilitation resource utilization.

### 4.4 Limitations and future directions

Despite these findings, this study has several limitations. First, this study used a cross-sectional design, which precludes causal inferences among the variables. In particular, the relationship between illness uncertainty and cardiac self-efficacy is likely to be bidirectional and reciprocal rather than strictly unidirectional. While high illness uncertainty may undermine patients' confidence in managing their cardiac condition, low cardiac self-efficacy may in turn intensify perceived uncertainty, as patients who doubt their ability to control symptoms or interpret bodily signals may find illness-related information more ambiguous and threatening (13, 57), thereby reinforcing a self-perpetuating cognitive cycle. The mediational ordering tested here was therefore based on theoretical reasoning rather than temporal precedence. Future studies should adopt longitudinal or cross-lagged designs with multiple measurement points to disentangle the temporal and potentially reciprocal relationships among these constructs.

Second, convenience sampling was used to collect data from three hospitals in Shanxi Province, which may limit the representativeness of the sample in terms of geographic region and hospital level. Differences in healthcare resource allocation, health education levels, and cultural background across regions may influence patients' experience of uncertainty in illness and their rehabilitation behavior patterns. Future research should expand the sampling scope, include patients with CHD from different regions and healthcare settings, and employ multicenter designs to improve the external validity and generalizability of the findings.

Third, all variables in this study were measured self-report questionnaires. Although Harman's single-factor test was conducted as a preliminary diagnostic assessment, the findings regarding common-method bias should be interpreted cautiously. Harman's single-factor test does not provide definitive evidence that common-method bias is absent, particularly in studies using same-source and same-time self-report measures. In the present study, uncertainty in illness, cardiac self-efficacy, fear of activity, and cardiac rehabilitation adherence were all measured using self-report questionnaires administered to the same participants during the same survey period. Therefore, shared method variance may have inflated some of the observed associations. Future studies should use longitudinal or multi-wave designs, collect data from multiple sources, incorporate objective indicators of cardiac rehabilitation adherence where possible, and consider statistical approaches such as latent method factor models to better evaluate and control for common-method bias.

In addition, two aspects of the Cardiac Self-Efficacy Scale should be noted. First, we administered a 13-item version that retained the spousal-relationship item excluded from the standard 12-item Chinese validation (38); although this item was fully answered under our on-site administration procedure, the resulting three-dimensional configuration was not independently re-validated in the present sample. Second, a 1–5 response format harmonized with the other instruments was used instead of the original 0–4 metric. While neither modification affects the relative associations or mediation pathways examined here (which rest on the covariance structure rather than absolute scores), both limit the direct comparability of absolute cardiac self-efficacy scores with prior studies. Future work should re-examine the factor structure and scoring of the scale in independent CHD samples.

A primary limitation concerns the measurement of cardiac rehabilitation adherence. The instrument used assesses patients' willingness to adhere across five dimensions rather than their objectively recorded behavior, and self-report measures may be subject to social desirability bias. We adopted this approach deliberately for two reasons. First, cardiac rehabilitation adherence is a multidimensional construct that extends well beyond attendance at supervised sessions to include home-based exercise, medication use, risk-factor management, nutritional management, and psychological self-management—behaviors that predominantly occur outside the clinical setting and for which no objective attendance or prescription records exist. Objective center-based logs would therefore capture only a narrow facet of adherence and would provide a less complete index of the construct than the validated multidimensional scale employed here. Second, adherence willingness/intention is a theoretically meaningful outcome and a recognized proximal determinant of actual adherence behavior in major health-behavior models, making it an appropriate criterion variable within our psychosocial framework, in which predictors and outcome are situated at the same individual, self-perceived level. To reduce social desirability bias, data were collected anonymously by research team members not involved in the patients' clinical care. Nonetheless, willingness does not equate to enacted behavior, and future studies should triangulate validated self-report with objective indicators (e.g., standardized attendance logs, wearable-device activity data) to capture both intention and actual adherence.

The primary LPA was based on 20 item-level indicators. Although this preserves within-dimension response heterogeneity, high-dimensional modeling with a moderate sample may increase sensitivity to starting values, and the high inter-item correlations may introduce local dependence that could affect class boundaries. We mitigated these concerns by using a large number of random starts (with replication of the best log-likelihood) and by conducting a sensitivity analysis with dimension-level scores, which reproduced the class solution. Future studies with larger samples would nonetheless allow more stable estimation of item-level profile models.

Although this study examined the chain mediating roles of cardiac self-efficacy and fear of activity, the direct effect of uncertainty in illness on cardiac rehabilitation adherence still accounted for 59% of the total effect, suggesting that other important mediating variables remain unexamined. Future research may further investigate the roles of potential mediators or moderators such as social support, health literacy, coping styles, and the quality of the physician-patient relationship to construct a more comprehensive theoretical model.

## 5 Conclusion

This cross-sectional study examined the association between uncertainty in illness and cardiac rehabilitation adherence among patients with CHD and explored theoretically informed indirect psychological pathways. Uncertainty in illness was significantly and negatively associated with cardiac rehabilitation adherence. Cardiac self-efficacy and fear of activity were statistically involved in separate and sequential indirect associations linking uncertainty in illness with cardiac rehabilitation adherence. At the person-centered level, uncertainty in illness showed significant heterogeneity and could be classified into three latent subgroups: cognitively clear–low uncertainty, partially informed–moderate uncertainty, and cognitively ambiguous–high uncertainty. These subgroups differed significantly in cardiac self-efficacy, fear of activity, and cardiac rehabilitation adherence, with the cognitively ambiguous–high uncertainty group showing the least favorable profile. These findings provide a theoretical basis for developing precise and stratified cardiac rehabilitation interventions tailored to different subgroups of patients with CHD. However, causal interpretations and intervention effects should be confirmed in future longitudinal and experimental studies.

## Data Availability

The original contributions presented in the study are included in the article/Supplementary Material, further inquiries can be directed to the corresponding author.
